# In-Situ Estimation of Soil Water Retention Curve in Silt Loam and Loamy Sand Soils at Different Soil Depths

**DOI:** 10.3390/s21020447

**Published:** 2021-01-10

**Authors:** Reem Zeitoun, Mark Vandergeest, Hiteshkumar Bhogilal Vasava, Pedro Vitor Ferrari Machado, Sean Jordan, Gary Parkin, Claudia Wagner-Riddle, Asim Biswas

**Affiliations:** School of Environmental Sciences, University of Guelph, Guelph, ON N1G 2W1, Canada; rzeitoun@uoguelph.ca (R.Z.); vandergm@uoguelph.ca (M.V.); hvasava@uoguelph.ca (H.B.V.); pferrari@uoguelph.ca (P.V.F.M.); sjordan@uoguelph.ca (S.J.); gparkin@uoguelph.ca (G.P.); cwagnerr@uoguelph.ca (C.W.-R.)

**Keywords:** field capacity, matric potential, parametric models, permanent wilting, soil water content

## Abstract

The soil water retention curve (SWRC) shows the relationship between soil water (θ) and water potential (ψ) and provides fundamental information for quantifying and modeling soil water entry, storage, flow, and groundwater recharge processes. While traditionally it is measured in a laboratory through cumbersome and time-intensive methods, soil sensors measuring in-situ θ and ψ show strong potential to estimate in-situ SWRC. The objective of this study was to estimate in-situ SWRC at different depths under two different soil types by integrating measured θ and ψ using two commercial sensors: time-domain reflectometer (TDR) and dielectric field water potential (e.g., MPS-6) principles. Parametric models were used to quantify θ—ψ relationships at various depths and were compared to laboratory-measured SWRC. The results of the study show that combining TDR and MPS-6 sensors can be used to estimate plant-available water and SWRC, with a mean difference of −0.03 to 0.23 m^3^m^−3^ between the modeled data and laboratory data, which could be caused by the sensors’ lack of site-specific calibration or possible air entrapment of field soil. However, consistent trends (with magnitude differences) indicated the potential to use these sensors in estimating in-situ and dynamic SWRC at depths and provided a way forward in overcoming resource-intensive laboratory measurements.

## 1. Introduction

Soil water retention curve (SWRC), a relationship between soil water potential (ψ), and soil water content (θ) is critical for various applications in soil science, hydrogeology, and hydrology [1]. It is often estimated using basic soil properties such as texture, or a water retention function; it is fitted to experimental data and provides an accurate estimation of soil hydrology. The fitted functions are then used in various hydrological models for drought and flood risks [2], green infrastructure in catchment-scale flood risk management by enhancing understanding of hydrological processes like interception, ponding, and evapotranspiration [3], and to calculate plant-available water and to estimate crop water requirements to manage irrigation scheduling [4]. Irrigation scheduling is one of the main methods to improve water use efficiency, minimize crop water stress, maximize yields, reduce labor through less irrigation, hold surface runoff, reduce the loss of nutrients through leaching into the groundwater, and promote water conservation in farms and agricultural fields. While water-saving irrigation methods like sprinkler or drip can improve applied water use efficiency, irrigation scheduling approaches are attractive methods in agriculture due to their enormous potential to save water [5]. Field evidence across China showed that irrigation scheduling practices can reduce irrigation water consumption of maize by 9–21% [6,7]. In another study in the United States, simple irrigation scheduling limited deep seepage to less than 5% and achieved 95% irrigation efficiency [8]. Different methods of irrigation scheduling include the hand feel method, electrical resistance blocks, and the water budget approach [9]. However, these methods are always associated with some drawbacks such as low accuracies of the hand feel method and its labor-intensive fieldwork, interference of soil salinity of resistance blocks, and periodic adjustments of the water budget approach [9]. The soil water regime approach based on sensors’ measurements of soil water content and soil water potential shows promise in determining irrigation schedules due to their minimal soil interference and real-time continuous soil measurements.

Water retention and availability in soil vary with its properties. The soil pores hold water with different degrees of tenacity, depending on the size of the pores and the amount of water in the soil. Soil water makes up the soil solution which is important in supplying the plants with essential nutrients [10]. Growing plants remove some of the moisture in the soil, and the rest remains either in the tiny pores or as thin films around the soil particles [10]. Soil hydraulic properties used to characterize the SWRC may include soil water pressure head, volumetric water content, and hydraulic conductivity [11]. With its vast importance, the SWRC is generally determined following traditional and well-accepted laboratory methods using hanging columns, pressure/suction table/cells, or pressure plates [12,13,14]. Although specific data points are occasionally used to characterize water retention curves, parametric models are preferred to provide the estimation and to describe the hydraulic relations for near-surface soils. The demand for these models is driven by their wide usage in mass transport and fluid flow and the increasing availability of simulation models [15]. An array of semi-empirical or empirical models have been revised and developed to fit discrete measured data [16,17]. Some of the models that showed feasibility for various kinds of soils include van Genuchten, Groenevelt–Grant, Campbell, and Kosugi [15,18,19,20,21,22]. The applicability of each model is restricted by its specific curve-shape and the soil texture [23]. For example, a previous study by Roy et al. showed that silt clay soil showed a poor match to the SWRC, derived by van Genuchten model, while silt and sandy loam soils had the best-fitted SWRCs [24]. Therefore, model comparison and selection are prerequisites to select the most suitable SWRCs for specific soils.

Measurements of θ and ψ, used in deriving SWRC, are traditionally determined by laboratory methods such as applying suction by a hanging-water-column and applying pressure above soil sample using pressure plates [25,26]. These methods involve several days of laboratory work, can be quite costly, represent a small section of the soil profile collected as cores [27], and require multiple repetitions of the same lengthy tests to produce comprehensive information. In contrast, an alternative method based on in-situ sensors’ measurements through the soil profile could provide an alternative method to derive SWRC. Developments in soil water sensor systems have allowed real-time continuous soil water measurement. Sensor systems can record soil water data which can be downloaded wirelessly within a certain radio range making the data acquisition easier for growers [28]. Some examples of inexpensive sensors used to measure soil water content and potential are capacitance-based sensors, resistivity-based granular matrix sensors, and tensiometers [5,29,30,31,32]. These sensors have been used in a variety of soil applications such as capturing soil water trends, estimating hydraulic properties in different soil textures, and sensor characterization in irrigated soil [5,33,34].

The overall objective of this study was to examine the feasibility of using sensor-measured soil hydraulic properties to estimate SWRC in-situ and in real-time. More specifically, this study was conducted to (i) examine the feasibility of time-domain reflectometry (TDR) and dielectric soil water potential sensors in characterizing dynamic soil hydraulic properties, (ii) use spreadsheet-optimized parametric models to derive SWRC from numerous measurements of soil tension, (iii) verify the relationship between the laboratory and field-measured SWRC, and (iv) estimate the plant-available water at different depths and soil types. To do so, we have combined and integrated measurements from TDR principle-based soil water measurement sensors (model TRIME PICO 32 from IMKO Inc. Ettlingen, BW, Germany) and dielectric principle-based soil water potential sensors (model MPS-6 from METER Inc. Pullman, WA, USA) to estimate in-situ and real-time SWRC and estimate the plant-available water. The in-situ and real-time soil water retention curves can provide affordable and easy ways in precision water management and irrigation. Three parametric modeling approaches were assessed in this study; van Genuchten, Kosugi, and Campbell [15,18,19,20,21] from multiple wetting–drying events in two different soils in a weighing lysimeter setting.

## 2. Materials and Methods

### 2.1. Sampling and Measurement Procedures

**Figure 1:**
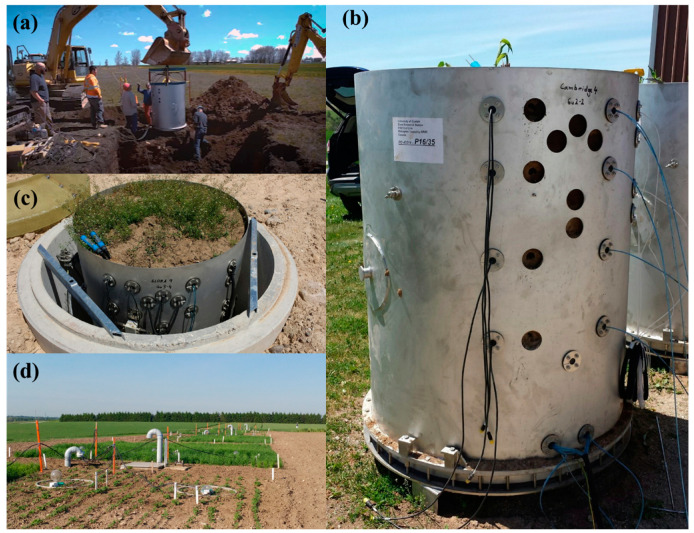
(**a**) Coring of undisturbed lysimeter core, (**b**) side view of lysimeter with sensors installed, (**c**) lysimeter lowered into well, and (**d**) the installed lysimeter setup within the research facility.

Two lysimeters that are metal cylinders with 1.50 m in depth and 1 m^2^ cross-sectional area were used to collect precipitation and evapotranspiration data. Using excavators, the lysimeters were slowly pressed down in the soil to allow for the preservation of column structure, and soil around the lysimeters was removed (Figure 1a). Once the lysimeters were filled, a hydraulic cutter was placed underneath them to slice through the soil at the bottom. The lysimeters were flipped upside down and ceramic cups were installed, to transmit water, before sealing the bottom. The lysimeters were then transmitted to the two monitoring study sites at Elora, Ontario and Cambridge, Ontario. Time-domain reflectometry-based soil water content sensors (model TRIME PICO 32 from IMKO, Inc. Ettlingen, BW, Germany) and dielectric principle-based water potential sensors (model MPS-6 from METER Inc. Pullman, WA, USA) were installed at two depths (5 and 30 cm) on the lysimeters (Figure 1b) and finally, the lysimeters were lowered into the wells (Figure 1c). Sensors’ data were collected at 10 min intervals during the month of October 2016 to estimate in-situ and dynamic SWRC. A total of 6 evapotranspiration events were observed within the month of October. Evapotranspiration events were selected to avoid the influence of hysteresis on the estimated SWRC. However, one of the events was discarded from the analysis due to the very short time cycle. The lysimeter measured data (ψ and θ) for each event were plotted and compared with a laboratory-derived SWRC, measured using conventional pressure plate methods from representative soil cores taken from the same depths as the soil sensor placement.

**Figure 2:**
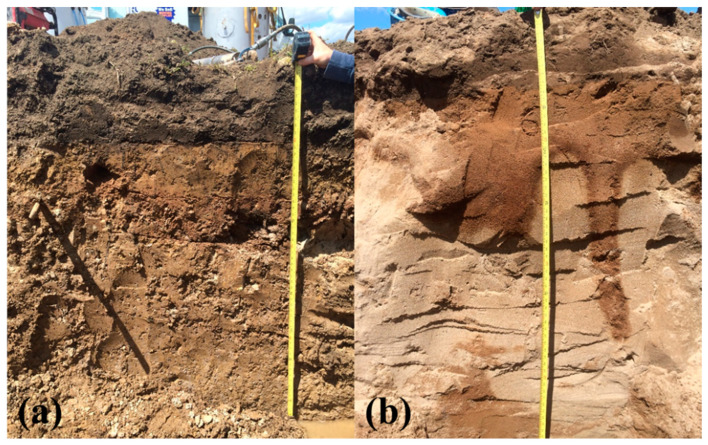
The soil profiles of (**a**) Elora, Ontario soil and (**b**) Cambridge, Ontario soil.

Soil profiles, classification information, and analysis were reported for both lysimeters’ soils (Figure 2 and Table 1). Soil horizons were identified according to the Canadian System of Soil Classification [35]. The soil texture analysis was performed on 3 cores for each of the observed horizons following the guidelines of Kroetsch et al. [36] (Table 1). The two lysimeters’ soils (extracted from Elora, Ontario and Cambridge, Ontario locations) were categorized as silt loam (classified as grey-brown luvisol [36]) and loamy sand (classified as brunisolic grey-brown luvisol [36]) (Table 1). Soil bulk density was identified according to the guidelines of Hao et al. [37] using the average of 9 measurements per depth and variation was assessed using standard deviation (Table 1).

### 2.2. Soil Water Characteristic Functions

Three models were selected to describe the relationship between ψ and θ. Among the most commonly used SWRC models is the parametric model of van Genuchten [18,19] which can be written as:

(1) $$\theta =\theta_{r}+\left(\theta_{s}-{\;\theta }_{r}\right){\left(1+{\left(-\propto \cdot \psi \right)}^{n}\right)}^{\frac{1}{n}-1}$$

where θ_r_ is the residual water content, θ_s_ is the saturated water content, ∝ (L^−1^) is a parameter (∝ > 0) to scale the matric head, ψ is the matric head, and n is a dimensionless parameter [38]. The van Genuchten model can be differentiated twice with respect to ψ to obtain the matric head at the inflection point (ψ_i_) given by Equation (2). Inverting this equation with respect to ∝ yields another expression for the SWRC called Kosugi model [15] which is given in Equation (3):

(2) ψi=m1−m∝

(3) $$\theta =\theta_{r}+\left(\theta_{s}-{\;\theta }_{r}\right){\left(1+m\cdot \frac{\Psi^{n}}{\Psi_{i}}\right)}^{\frac{1}{n}-1}$$

The other retention model used in this study was derived by Campbell [20]; it assumes θ_r_ is equal to zero and can be written as:

(4) $$\theta =\theta_{s}-\;{\left(\frac{\psi_{\mathrm{ma}}}{\psi }\right)}^{\lambda }$$

where λ is a dimensionless parameter that characterizes the pore-size distribution, and ψ_ma_ is the air-entry potential.

### 2.3. Methodology for Fitting Water Retention Parametric Models

Equations (1), (3), and (4) were fitted to the θ and ψ data gathered from the TDR and MPS-6 sensors, respectively, with a non-linear least-square fitting method. The models were entered into a spreadsheet (Microsoft Office 365 Excel) with the measured ψ being a constant. The sum of the squared difference between the measured and modeled θ was determined and the spreadsheet extension solver was used to minimize this value by changing the model parameters. Through this method, the models were optimized and values for θ_r_, θ_s,_ and all other parameters in each model were determined. The retention curves for each model of the 5 events in each lysimeter were plotted together and then the model parameters for the 5 events for each lysimeter were averaged and used to extrapolate the data to determine the field capacity (FC) and the permanent wilting point (PWP) in the soil.

### 2.4. Plant-Available Water Calculations

The van Genuchten model was used to calculate plant-available water (PAW). This was calculated as the difference between FC and PWP (at soil matric potential of −0.33 bar and −15 bars, respectively), multiplied by the soil depth (5 cm and 30 cm), Equation (5).

(5) PAW=(FC−PWP) ×soil depth

### 2.5. Descriptive Statistics

The mean, minimum, maximum, and standard deviation (σ) of θ and ψ data for the two soil types at two depths were reported for 5 events. The fitting performance of each SWRC model was assessed based on root mean square error (RMSE) and the coefficient of determination (R^2^).

(6) $$\mathrm{RMSE}=\;\sqrt{\overset{i}{\underset{n=1}{\sum }}\;\frac{{\left(\theta_{i}-\theta_{i,f}\right)}^{2}}{n}}$$

(7) $$R^{2}=1-\;\frac{\sum_{n=1}^{i}\;{\left(\theta_{i}-\theta_{i,f}\right)}^{2}}{\sum_{n=1}^{i}\;{\left(\theta_{i}-\theta_{\mathrm{avg}}\right)}^{2}}$$

where n is the number of soil water retention data points collected in each event, θ_i_ and θ_i,f_ are the measured and the fitted soil water content, and θ_avg_ is the mean of measured soil water content.

The best fit model was the one with the least RMSE and the highest R^2^ and closest to unity. To test the sensor’s performance, laboratory-derived soil water volumetric content (θ_L_) from representative soil cores at 5 cm and 30 cm soil depths were compared to the field sensors θ at the same soil depths. The correlation coefficient (r_L_) and the overall error (RMSE_L_) between θ_L_ and the parametric models fitted soil water content (θ_f_) were evaluated. The intercept (b) and slope (m) of the linear regression equations were also reported. The mean difference (Md) was used to evaluate the difference between the averaged field measured water content, θ_avg,_ and the averaged laboratory-derived soil water volumetric content, θ_L,avg_. The relative standard deviation (RSD) of the average van Genuchten, Kosugi, and Campell model parameters for 5 distinct periods of evapotranspiration was used to evaluate the variation of the parameters in silt loam and loamy sand soils at two soil depths.

(8) $$\theta_{f}={m\theta }_{L}+b$$

(9) $${\mathrm{RMSE}}_{L}=\;\sqrt{\overset{i}{\underset{n=1}{\sum }}\;\frac{{\left(\theta_{L}-\theta_{i,f}\right)}^{2}}{n}}$$

(10) $$\mathrm{Md}={\;\theta }_{\mathrm{avg}}-{\;\theta }_{L,\mathrm{avg}},$$

(11) $$\mathrm{RSD}=\;\frac{\sigma_{\mathrm{Parameter}\;\mathrm{Ev}1-\mathrm{Ev}6}}{{\mathrm{Average}\;\mathrm{parameter}}_{\mathrm{Ev}1-\mathrm{Ev}6}}\;\times 100,$$

## 3. Results and Discussion

### 3.1. Field Data SWRC at Multiple Evapotranspiration Events

**Figure 3:**
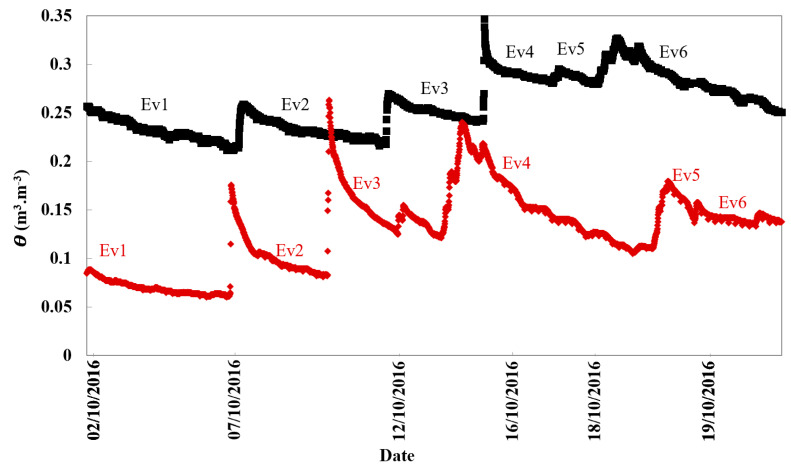
Soil volumetric water content measured by time-domain reflectometer (TDR) sensors in October for Elora silt loam soil (black) and Cambridge loamy sand soil (red) at 5 cm depth, reflecting the rainfall and evapotranspiration events (Ev1–6).

**Figure 4:**
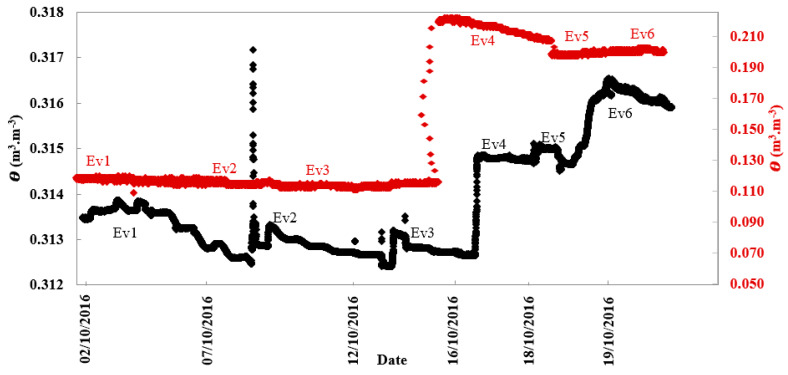
Soil volumetric water content measured by TDR sensors in October for Elora silt loam soil (black) and Cambridge loamy sand soil (red) at 30 cm depth, reflecting the rainfall and evapotranspiration events (Ev1–6).

The θ data measured by the TDR sensors in Elora and Cambridge soils are presented in Figure 3 and Figure 4, which indicate that the sensors captured all of the evapotranspiration events (Ev1–Ev6) occurring in October. Ev5 was discarded from the analysis due to the very short time cycle. Loamy sand (Figure 3 and Figure 4, red) showed remarkably lower θ at all rainfall and subsequent evapotranspiration events. This can be explained by the effect of soil texture in holding less water. The large surface area of the soil’s smaller particles, such as silt, allows the soil to hold more water [10]. Hence, silt loam soil exhibited higher θ values at both depths (Figure 3 and Figure 4, black) than loamy sand soils (Figure 3 and Figure 4, red).

Both soils showed different and in most cases less variable θ values at deeper soil depths (Figure 4) than at shallower soil depths (Figure 3). Deeper soil layers often showed more consistent θ values due to the damping effects of the overlying soil. However, there was a marked jump in θ measured in the Cambridge soil at 30-cm, but only during the evapotranspiration event on October 16.

**Figure 5:**
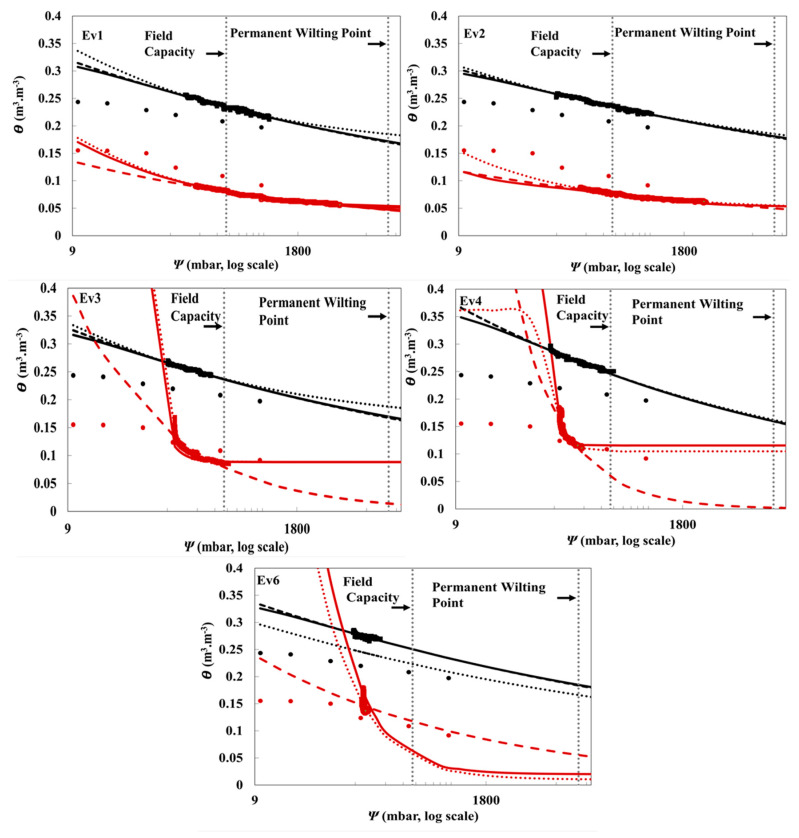
Soil water retention curves (SWRCs) (ψ data from MPS-6 and θ from TDR) of silt loam (black) and loamy sand (red) soils measured by two lysimeters (one each) at 5 cm depth. The graphs illustrate the scattering of parametric models derived from the field data of 5 evapotranspiration events (Ev1, Ev2, Ev3, Ev4, and Ev6) compared with the laboratory-measured data, θ_L_. θ_L_ (•), field data (■), Kosugi (**…**), van Genuchten (▬), Campbell (**---**).

**Figure 6:**
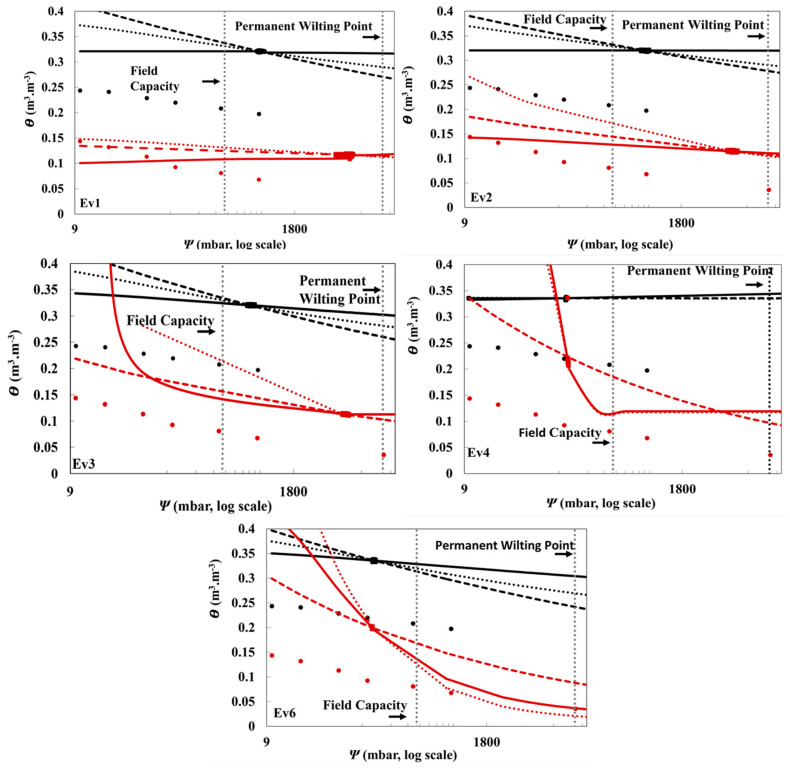
SWRCs (ψ data from MPS-6 and θ from TDR) of silt loam (black) and loamy sand (red) soils measured by two lysimeters (one each) at 30 cm depth. The graphs illustrate the scattering of parametric models derived from field data of 5 evapotranspiration events (Ev1, Ev2, Ev3, Ev4, and Ev6) compared with the laboratory-measured data, θ_L_. θ_L_ (•), field data (■), Kosugi (**…**), Van Genuchten (▬), Campbell (**---**).

A comparison among field-measured, laboratory-measured, and model-fitted θ is presented in Figure 5 and Figure 6 for five evapotranspiration events of silt loam and loamy sand soils at 5 and 30 cm depths. The highly precise and frequent measurement intervals of the TDR and MPS sensors enabled a greater number of data points to be used than were used for the laboratory data (Figure 5 and Figure 6). The field data for silt loam soil appear to show consistently greater θ than laboratory data at both depths, whereas, the differences between lab and field data for loamy sand soil are less consistent. The fitted models had slightly different parameters for each event, showing real-time trends of drying events and the dynamic nature of these models (Table 2). A similar study was conducted by Steenpass et al. to provide real-time continuous soil water data by integrating TDR measurements with surface soil temperature measurements [27]. Steenpass et al. measured the changes in surface soil temperature and used them to derive the soil moisture content. In their method, a layered soil profile was used to derive soil hydraulic properties from the real data, which consequently resulted in many uncertainties. Their experiment used TDR probe measurements to establish initial soil water conditions as well as using the derivation from the surface soil temperature data. The estimated SWRCs were found to be quite similar to the laboratory results of their deep soil cores, but the retention curves from the surface varied strongly from the lab data [27].

The averaged model parameters for loamy sand soil showed high RSD values (Table 3). This indicated the high variations within the parameters at different evapotranspiration events. On the other hand, silt loam soils showed lower RSD values indicating the low variations within the model parameters at different evapotranspiration events. Loamy sand soils can be recharged with soil moisture at a faster rate than soils with finer texture such as silt loam [10]. However, they are less capable of holding as much water as silt loam [39]. The high recharging and drainage ability of loamy sand soils could explain why they have a highly dynamic nature as expressed by the high variations of the RSD values of the model parameters.

The variation of θ and ψ values in Table 4 shows the dynamic behavior of the multiple wetting and drying events (Ev1–Ev6). Considering the variation of θ and ψ values within the 5 events (Table 4), Ev1–Ev2 were noticeably drier events than Ev3–Ev6. Soil texture also affected the θ and ψ values, with silt loam soils having higher θ values. Loamy sand soils have a higher bulk density than silt soils at 0–30 cm soil depth (Table 1). This makes silt soils have more pore space and a higher capacity to hold water. Deeper soil samples (30 cm) showed higher θ values than shallower soil samples (5 cm) (Table 4) due to the higher porosity of deeper soil layers [10].

### 3.2. Fitting Performance of van Genuchten, Kosugi, and Campbell Parametric Models

The three models delivered high R^2^, close to unity, and small RMSE for Ev1–Ev4 for both soils at 5 cm depth (Table 5). The models generally provided a better fit for the silt loam data than the loamy sand (Table 5). However, Ev6 at 5 cm depth and Ev1–Ev6 at 30 cm depth for both soil types registered low R^2^ (Table 5). This can be explained by the high proximity of the data points at these events (Figure 5 Ev6 and Figure 6 Ev1–Ev6) which affected the fitting performance of SWRCs models.

### 3.3. Sensor Performance

**Figure 7:**
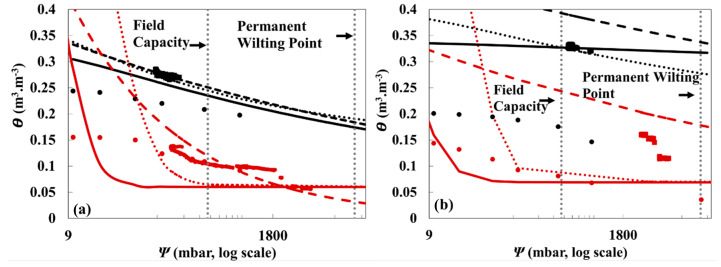
SWRC models, adjusted to averaged field data, (ψ data from MPS-6 and θ from TDR) of silt loam (black) and loamy sand (red) soils measured by two lysimeters (one each) at (**a**) 5 cm and (**b**) 30 cm depths. The graphs illustrate the scattering of averaged parametric models derived from field data of 5 evapotranspiration events compared with the laboratory-measured data, θ_L_. θ_L_ (•), field data (■), Kosugi (**…**), Van Genuchten (▬), Campbell (**---**).

The averaged parametric models (Figure 7) were used to determine the relationship between θ (sensors data) and θ_L_ (laboratory driven data) to assess the overall performance of sensors at different conditions. At shallow soil depth (5 cm), the difference between θ and θ_L_ decreased as ψ increased (Figure 7). The large differences at lower ψ could be because of air entrapment in field soils as data was collected under dynamic as opposed to static conditions for the laboratory data. The water infiltration rate, the speed at which water enters the soil, is always controlled by the rate of air outflow. Water movement in the vadose zone takes place because of immiscible displacement between air and water [40]. As ψ increases, the soil will have less θ i.e., more air outflow is possible and less entrapped air.

The estimated water content of the field data and parametric models were not very close to the data collected in the laboratory and overestimated the soil water content at different depths, which is reflected by the positive Md values exhibited by most of the SWRC models (Table 6). Silt loam soil registered higher r_L_ than loamy sand soil (Table 6). Loamy sand has a higher permeability than silt loam (2x more) [41]. More permeability results in more water inflow and more displacement between air and water within the soil, which could affect the sensors’ readings, causing a consistent variation between the dynamic field modeled data and the static laboratory data at various depths and soil tensions (Figure 5, Figure 6 and Figure 7), which results in smaller r_L_ relative to silt loam (Table 6). The silt loam soil exhibited lower RMSE_L_ and Md values at lower depth (5 cm) than at higher depths (30 cm). With high air pressure at higher depths (30 cm) in the silt loam soil, high air outflow will take place in the soil, making a larger variation between the laboratory data and the modeled data, which is reflected by the Md and RMSE_L_ values (Table 6). The slope (m) and intercept (b) values of the linear regression equations (Table 6) showed that site-specific calibration could enhance the sensors’ precision greatly. In a similar study, tensiometer and electrical resistance sensors were used to measure ψ and θ, respectively, in a pecan field in Texas, USA [5]. Both sensors underestimated the soil water content at different depths but registered a high coefficient of determination (R^2^ = 0.71) between the sensor water content and the soil gravimetric water under field conditions. Overall the sensors managed to capture the general trends of soil water content; however, soil sensors, with factory calibration only, had levels that were lower than the ideal level [5].

### 3.4. Plant-Available Water

PWP and FC are two important hydraulic features in soil that determine the moisture content at which the plant cannot absorb water and the point at which water moves slowly after irrigation, respectively [42]. FC and PWP are widely used for scheduling irrigation and assessing the plants’ water requirements. From Figure 5 and Figure 6, FC and PWP can be estimated at a soil matric potential of −0.33 bar and −15 bars respectively [42], and the difference in θ between these two points was used to calculate PAW [43]. The van Genuchten model was used to calculate PAW since it is the most used model in water retention studies, and it registered small RMSE values between θ and θ_L_ (Table 6). The variations in PAW values for Ev1–Ev6 for both soils (Table 7) reflect again the dynamic nature of these data. The PAW estimated for silt loam soil at 5-cm depth were the most consistent between evapotranspiration events. The SWRC models, adjusted to averaged field data, were used to calculate PAW values for the silt loam at 5 and 30 cm depth, which were equal to 0.292 and 0.288 cm, respectively. The SWRC models, adjusted to averaged field data, of loamy sand showed comparatively very low PAW at both depths; 0.10 × 10^−4^ cm (5 cm depth) and 0.10 × 10^−2^ cm (30 cm depth) which can be explained by the high permeability of loamy sand which decreases the amount of available water to the plant [44].

## 4. Conclusions

In conclusion, integrating TDR and MPS-6 sensors introduces a relevant method to describe the in-situ conditions of the soil. Simple water content may not provide a lot of information, however, TDR and MPS data can be used to estimate plant-available water in-situ and in real-time. The resulting averaged models showed a strong correlation between the laboratory-measured data and the modeled data with the van Genuchten parametric model being the closest to the laboratory data and Campbell parametric model showing the best fitting performance towards the field measured data. However, the reported positive Md values between θ and θ_L_ indicate the overestimation of the soil water content. A possible explanation of the differences between the laboratory-measured data and the field-modeled data could be air entrapment or air outflow/inflow in the field soil collected under dynamic conditions, differences in lab and field samples bulk density, or the sensors’ lack of site-specific calibration. The soil texture or other soil properties such as available air at different depths, pH, conductivity, and nutrient concentrations could affect the sensors’ performance. Calibrating the soil water sensors against these properties is a recommended enhancement towards more accurate sensor results. Incorporating soil properties that have temporal changes on soil could also be a helpful solution to improve the sensors’ reliability. Future research on the improved soil properties on SWRC estimation using TDR and MPS-6 sensors is recommended, especially using different soil textures and a wider range of soil water content conditions. Using real-time soil tension data with spreadsheet optimized parametric models to derive SWRCs is a promising technique that can be used to reflect the dynamic nature of the soil drying events to deduce the plant available water, providing better irrigation management for different soil types. Measurements of soil–water status are crucial in irrigation scheduling, especially in soils with a narrow PAW range and low soil-water holding capacity such as loamy sand.

## Figures and Tables

**Table 1:** Soil identification information.

	Soil Type					
Soil Property	Elora, Ontario Soil			Cambridge, Ontario Soil		
Mineral soil horizons	Ap	Bt	Ck	Ap	Btj	Ck
Horizon depth (cm)	0–32 *	32–61 ^#^	61^+^	0–28 **^§^**	28–55 ^ʈ^	55 ^+^
Horizon thickness (cm)	20–34 *	20–30 ^#^	-	20–31 **^§^**	15–90 ^ʈ^	-
Sand (%)	38.0 ^¶^	44.7 ^¶^	49.4 ^¶^	79.2 ^‡^	82.0 ^‡^	88.8 ^‡^
Silt (%)	54.5 ^¶^	40.3 ^¶^	38.1 ^¶^	17.5 ^‡^	13 ^‡^	8.7 ^‡^
Clay (%)	7.5 ^¶^	15.0 ^¶^	12.5 ^¶^	3.3 ^‡^	5.0 ^‡^	2.5 ^‡^
Textural class	silt loam	loam	loam	loamy sand	loamy sand	sand
Bulk density (g cm^−3^)	1.53 ± 0.12	1.71 ± 0.08	1.78 ± 0.11	1.71 ± 0.11	1.68 ± 0.09	1.64 ± 0.07

* horizon boundary distinctness Ap/Bt is clear (2–5 cm) and form is smooth; ^#^ horizon boundary distinctness B/Ck is gradual (5–15 cm) and the form is smooth; ^¶^ average particle size distribution of 3 control sections; ^‡^ average particle size distribution of 2 control sections; ^§^ horizon boundary distinctness Ap/B is clear (2–5 cm) and form is smooth; ^ʈ^ horizon boundary distinctness B/Ck is abrupt (<2 cm) and the form is irregular (depth greater than width).

**Table 2:** Summary of van Genuchten, Kosugi, and Campbell model parameters for 5 distinct periods of evapotranspiration in silt loam and loamy sand soils at 5 and 30 cm depths.

Event#	Van Genuchuten				Kosugi				Campbell		
	θ_r_ (m^3^m^−3^)	θ_s_ (m^3^m^−3^)	∝ (cm^−1^)	n	θ_r_ (m^3^m^−3^)	θ_s_ (m^3^m^−3^)	ψ_i_ (mbar)	n	ψ_ma_ (mbar)	θ_s_ (m^3^m^−3^)	λ
Silt loam (5 cm)											
Ev1	0.04	0.33	0.16	1.10	0.15	0.42	0.61	1.24	0.03	0.51	0.08
Ev2	0.04	0.32	0.17	1.09	0.10	0.43	0.03	1.12	0.02	0.46	0.07
Ev3	0.04	0.34	0.15	1.11	0.15	0.41	0.58	1.22	0.03	0.55	0.09
Ev4	0.04	0.32	0.17	1.04	0.14	0.42	0.59	1.18	0.03	0.54	0.08
Ev6	0.04	0.35	0.17	1.10	0.14	0.39	0.58	1.18	0.03	0.54	0.08
Loamy sand (5 cm)											
Ev1	0.04	0.27	0.48	1.35	0.04	0.25	1.65	1.40	0.03	0.31	0.14
Ev2	0.05	0.13	0.06	1.34	0.05	0.51	0.05	1.36	0.10	0.20	0.12
Ev3	0.09	0.99	0.02	4.50	0.09	0.99	46.15	4.50	1.29	0.99	0.45
Ev4	0.12	0.52	0.01	9.26	0.10	0.36	76.21	5.89	12.40	0.99	0.86
Ev6	0.02	0.99	0.04	2.26	0.01	0.99	12.28	2.05	0.04	0.70	0.20
Silt loam (30 cm)											
Ev1	0.04	0.33	0.16	1.00	0.16	0.39	0.60	1.07	0.03	0.58	0.04
Ev2	0.04	0.32	0.17	1.00	0.10	0.41	0.03	1.05	0.02	0.52	0.04
Ev3	0.04	0.35	0.15	1.02	0.16	0.40	0.59	1.09	0.03	0.61	0.04
Ev4	0.04	0.31	0.17	0.99	0.14	0.41	0.59	1.12	0.03	0.55	0.04
Ev6	0.04	0.36	0.17	1.02	0.14	0.39	0.58	1.09	0.03	0.59	0.04
Loamy sand (30 cm)											
Ev1	0.04	0.10	0.60	0.96	0.07	0.15	1.69	1.09	0.02	0.15	0.04
Ev2	0.05	0.15	0.06	1.07	0.05	0.48	0.05	1.19	0.10	0.26	0.05
Ev3	0.11	0.50	0.03	4.48	0.11	0.99	30.81	4.47	0.19	0.33	0.11
Ev4	0.12	0.51	0.01	8.52	0.12	0.47	96.23	8.68	0.04	0.86	0.17
Ev6	0.02	0.49	0.06	1.49	0.01	0.86	5.01	1.59	0.04	0.75	0.02

**Table 3:** Summary of averaged van Genuchten, Kosugi, and Campell model parameters for 5 distinct periods of evapotranspiration in silt loam and loamy sand soils at 5 and 30 cm depths.

Soil Type (Depth)	Model Parameters							
	van Genuchuten							
	θ_r_ (m^3^m^−3^)	RSD (%)	θ_s_ (m^3^m^−3^)	RSD (%)	∝ (cm^−1^)	RSD (%)	n	RSD (%)
Silt loam (5 cm)	0.04	0.00	0.33	3.93	0.16	5.45	1.09	2.55
Loamy sand (5 cm)	0.06	64.08	0.58	68.88	0.12	164.79	3.74	89.34
Silt loam (30 cm)	0.04	0.00	0.33	6.21	0.16	5.45	1.01	1.33
Loamy sand (30 cm)	0.07	65.27	0.35	58.94	0.15	165.35	3.30	98.50
	Kosugi							
	θ_r_ (m^3^m^−3^)	RSD (%)	θ_s_ (m^3^m^−3^)	RSD (%)	Ψi (mbar)	RSD (%)	n	RSD (%)
Silt loam (5 cm)	0.14	15.25	0.41	3.66	0.48	52.46	1.19	3.88
Loamy sand (5 cm)	0.06	63.82	0.62	56.47	27.27	121.27	3.04	67.31
Silt loam (30 cm)	0.14	17.50	0.40	2.50	0.48	52.41	1.08	2.41
Loamy sand (30 cm)	0.07	62.42	0.59	57.04	26.76	152.47	3.40	95.78
	Campbell							
	ψ_ma_ (mbar)	RSD (%)	θ_s_ (m^3^m^−3^)	RSD (%)	λ	RSD (%)		
Silt loam (5 cm)	0.03	15.97	0.52	7.07	0.08	8.84		
Loamy sand (5 cm)	2.77	195.12	0.64	58.18	0.35	88.15		
Silt loam (30 cm)	0.03	15.97	0.57	6.20	0.04	0.00		
Loamy sand (30 cm)	0.08	89.01	0.47	67.00	0.08	78.72		

**Table 4:** Descriptive statistics of sensors ψ and θ data at 5 and 30 cm depths for silt loam and loamy sand soils, describing the variations of θ and ψ values within 5 evapotranspiration events.

Event#	ψ Mean (mbar)	ψ Max (mbar)	ψ Min (mbar)	σ * (mbar)	θ Mean (m^3^m^−3^)	θ Max (m^3^m^−3^)	θ Min (m^3^m^−3^)	σ * (m^3^m^−3^)
Silt loam (5 cm)								
Ev1	410	918	127	202	0.233	0.256	0.211	0.010
Ev2	403	847	51.0	180	0.234	0.259	0.221	0.009
Ev3	161	247	91	42.9	0.254	0.269	0.243	0.007
Ev4	144	360	82	63.9	0.273	0.298	0.250	0.012
Ev6	111	160	86	15.4	0.274	0.286	0.265	0.004
Loamy sand (5 cm)								
Ev1	1487	4845	162	1360	0.070	0.092	0.055	0.010
Ev2	1123	3000	157	859	0.070	0.088	0.059	0.007
Ev3	188	291	107	61.7	0.106	0.168	0.087	0.019
Ev4	120	171	100	20.2	0.142	0.184	0.110	0.020
Ev6	111	121	105	3.81	0.146	0.179	0.133	0.011
Silt loam (30 cm)								
Ev1	782	877	722	39.9	0.321	0.323	0.319	0.001
Ev2	739	815	102	52.4	0.319	0.322	0.249	0.005
Ev3	661	715	102	41.3	0.320	0.322	0.249	0.004
Ev4	107	111	10.0	4.22	0.335	0.338	0.249	0.004
Ev6	120	124	102	2.16	0.335	0.338	0.249	0.004
Loamy sand (30 cm)								
Ev1	6296	7424	4862	672	0.116	0.118	0.115	0.002
Ev2	6414	7107	5500	333	0.114	0.117	0.112	0.001
Ev3	6219	6704	5750	241	0.112	0.115	0.111	0.001
Ev4	112	113	111	0.799	0.215	0.336	0.207	0.006
Ev6	115	115	114	0.281	0.199	0.202	0.197	0.001

* σ is the standard deviation of sensors’ data for each event.

**Table 5:** Fitting performance of van Genuchten, Kosugi, and Campbell SWRT models using sensors ψ and θ data at 5 and 30 cm depths for silt loam and loamy sand soils.

	Van Genuchten		Kosugi		Campbell	
Event#	RMSE (%)	R^2^	RMSE (%)	R^2^	RMSE (%)	R^2^
Silt loam (5 cm)						
Ev1	0.19	0.97	0.18	0.97	0.19	0.97
Ev2	0.15	0.98	0.15	0.98	0.15	0.98
Ev3	0.12	0.97	0.11	0.97	0.12	0.97
Ev4	0.33	0.94	0.33	0.94	0.33	0.95
Ev6	0.21	0.68	3.01	0.68	0.21	0.68
Loamy sand (5 cm)						
Ev1	0.13	0.98	0.13	0.98	0.17	0.97
Ev2	0.11	0.97	0.11	0.98	0.17	0.95
Ev3	0.11	0.50	0.47	0.93	0.80	0.81
Ev4	0.95	0.84	1.02	0.82	1.10	0.74
Ev6	2.26	0.22	1.16	0.23	1.22	0.09
Silt loam (30 cm)						
Ev1	0.06	0.02	0.08	0.03	0.03	0.03
Ev2	0.61	0.42	0.71	0.42	0.75	0.43
Ev3	0.56	0.60	0.65	0.61	0.72	0.61
Ev4	0.45	0.00	0.45	0.00	0.45	0.00
Ev6	0.45	0.10	0.46	0.10	0.47	0.10
Loamy sand (30 cm)						
Ev1	0.10	0.21	0.15	0.35	0.14	0.34
Ev2	0.11	0.12	0.11	0.19	0.10	0.19
Ev3	0.07	0.98	0.07	0.98	0.15	0.99
Ev4	0.16	0.95	0.16	0.95	0.41	0.53
Ev6	0.14	0.11	0.28	0.11	0.52	0.11

**Table 6:** Statistical summary of sensor performance.

	Silt Loam					Loamy Sand				
SWRT Model	RMSE_L_ (%)	r_L_	Md (m^3^m^−3^)	m ^+^	b ^+^	RMSE_L_ (%)	r_L_	Md (m^3^m^−3^)	m ^+^	b ^+^
5 cm depth										
van Genuchten	4.78	0.99	0.05	1.65	−0.10	7.56	0.55	−0.03	1.84	−0.13
Kosugi	6.19	0.98	0.06	2.22	−0.22	24.5	0.85	0.14	7.70	−0.73
Campbell	5.79	0.99	0.05	1.95	−0.16	12.8	0.91	0.09	4.04	−0.30
30 cm depth										
van Genuchten	14.97	0.92	0.15	0.17	0.29	2.65	0.69	−0.02	0.61	0.03
Kosugi	16.48	0.92	0.16	1.10	0.19	0.11	0.86	0.15	5.05	−0.25
Campbell	23.06	0.94	0.23	1.19	0.14	2.01	0.99	0.17	1.27	0.13

^+^ m is the slope; b is the intercept.

**Table 7:** Summary of plant-available water (PAW) values derived using the van Genuchten model.

PAW (cm)				
**Event#**	Silt Loam (5 cm)	Loamy Sand (5 cm)	Silt Loam (30 cm)	Loamy Sand (30 cm)
Ev1	0.32	0.14	0.15	NA
Ev2	0.27	0.11	0.03	0.51
Ev3	0.34	0.28 × 10^−2^	0.66	0.39 × 10^−2^
Ev4	0.43	1.73 × 10^−5^	0.18	0.10 × 10^−2^
Ev6	0.33	0.18	0.75	2.75

## Data Availability

Derived data supporting the findings of this study are available from the corresponding author on request.
